# Supplementary material for: Vaccination experiences and decision-making in older adult Korean immigrants living in Canada: A qualitative descriptive study
Source: PLOS Glob Public Health. 2025 Dec 19;5(12):e0005538. doi: 10.1371/journal.pgph.0005538 (PMC12716682; doi:10.1371/journal.pgph.0005538)
Supplement: Attachment — Submitted filename: 2025_Vaccination in Korean immigrants_Response_251104.docx [file pgph.0005538.s002.docx]

PLOS Global Public Health

Manuscript title: Vaccination experiences and decision-making in older adult Korean immigrants living in Canada: A qualitative descriptive study

Authors: Kim J, Park I, Cha E, Sulis G, Yang S, Papenburg J, Li P, and Banerjee A

**Response to the Editor**

Dear Editor,

Thank you for your assessment of our manuscript, “Vaccination experiences and decision-making in older adult Korean immigrants living in Canada: A qualitative descriptive study”. Please see below for our point-by-point response to the reviewers.

**Reviewer 1**

Congratulations on your manuscript addressing an important and timely topic, as vaccine confidence and the reduction of inequities among migrant populations remain key global public health priorities. You have undertaken a rigorous and well-reported study. Your paper would be further strengthened by more clearly situating the findings within the existing body of literature and by providing more specific recommendations to guide future research.

Response: Thank you for your review of our submission. Please see below for our point-by-point response to your recommendations.

1. Intro (p.4): “…shingles vaccination is publicly funded in 4 public health jurisdictions, among which only one provides the vaccine for free as per NACI recommendations for adults aged 50 years and older” > I would suggest specifying the four jurisdictions and indicating which province offers the shingles vaccine for free.

Response: We have clarified the 4 jurisdictions that publicly fund shingles vaccinations and the one jurisdiction that provides it for free as per NACI recommendations in lines 76-80.

“Influenza and pneumococcal vaccinations are publicly funded for those aged 65 years and older across all provinces and territories in Canada, while shingles vaccination is publicly funded in Ontario, Quebec, Prince Edward Island, and Yukon, among which only Prince Edward Island provides the vaccine for free as per NACI recommendations for adults aged 50 years and older [14].”

2. Intro (p.4-5): “Studies also indicate that vaccination coverage in immigrants is lower by 2.4% for influenza, 10.7% for pneumococcal disease, and 3.8% for shingles, compared to non-immigrants…” > Including data on vaccination coverage among older Korean immigrants, if available, would strengthen this section.

Response: There are no data available on vaccination coverage among older Korean immigrants in Canada or USA. However, studies have reported vaccination coverage by ethnicity among older adults (one in Canada and USA each), which we have included in lines 102-112.

“According to a study conducted using 2003-2009 Canadian Community Health Survey data, Korean adults aged 65 years and older reported lower, although not statistically significant, coverage of influenza vaccination than those of other ethnic groups with the exception of Latin American, West Asian/Arab, and Black older adults [22]. This was the only study in the Canadian context that reported vaccination coverage among Korean older adults, but was not specific to Korean immigrant older adults. Similarly, a study conducted using 2009-2012 REACH US Risk Factor Survey reported that Korean American adults aged 50 years and older reported lower influenza vaccination coverage than those of other ethnic groups with the exception of Non-Hispanic Black adults, and that Korean adults aged 65 years and older reported lowest coverage of pneumococcal vaccination among population groups analyzed [23].”

3. Intro (p.5-6): “Studies on vaccine willingness and healthcare service use among Korean immigrants conducted in North America suggest that Korean immigrants…” > It would be important to situate your study within the existing evidence base and to clearly identify the specific gaps your research addresses. Are the relevant studies based in Canada or primarily in the U.S.? Do they focus on child vaccination or on older sub-populations? Are they centred on vaccine uptake specifically, or do they address broader patterns of healthcare service use?

Response: We have further clarified the studies on vaccine willingness and healthcare service use conducted in North America in lines 112-118.

“Other studies on vaccine willingness and healthcare service use among Korean adults in North America, primarily conducted in USA and focusing on COVID-19 vaccinations, suggest that Korean adults are less willing compared to other Asian adults to get vaccinated and less likely to access and utilize healthcare services, and that various barriers negatively impact Korean immigrants’ health and healthcare utilization, such as lack of social support, language difficulties, geographic constraints, and economic challenges [19,24–26].”

4. Intro (p.6): “…there is a pressing need to better understand the decision-making processes of Korean immigrants, to inform tailored strategies to improve vaccine uptake and hesitancy.” > Is there evidence indicating not only lower vaccination coverage rates but also a higher risk of influenza, shingles, or pneumococcal disease among older Korean immigrants, which would further underscore the urgency of the issue?

Response: Unfortunately, there are no data on the burden of influenza, pneumococcal, or shingles diseases among Korean older adult immigrants. We have clarified this in lines 119-124.

“While data on the burden of vaccine-preventable diseases on Korean older adult immigrants are not available, given that NACI recommends older adults receive influenza, pneumococcal, and shingles vaccines [11–13], but that Korean immigrants have lower vaccination willingness and healthcare service use [19,24–26], there is a pressing need to better understand the decision-making processes of Korean immigrants, to inform tailored strategies to improve vaccine uptake and hesitancy.”

5. Methods: I would suggest introducing Bronfenbrenner’s socio-ecological model (SEM) in the methods section, rather than at the beginning of the results section, along with a brief presentation of the SEM categories.

Response: We moved the introductory paragraph on SEM to the methods section, in lines 213-236.

“We organized emerging themes and sub-themes using the socio-ecological model (SEM). Introduced by Bronfenbrenner, the framework demonstrates that personal and environmental factors interact and intersect to influence one’s health and health behaviours [41,42]. We chose to present our results using the SEM over more vaccination-specific frameworks such as the World Health Organization’s behavioural and social drivers of vaccination [16], which was conceptualized and developed using the SEM [43], to highlight the interaction of factors across different layers in shaping vaccination decision-making, as well as the importance of higher-level, more structural and systemic factors that impact vaccination decision-making, partially through downstream effects on individual-level determinants of vaccination. Notably, the domains in the BeSD of vaccination are related to and reflect various SEM levels [44]. For instance, the thinking and feeling domain corresponds to the intrapersonal level, whereas the social processes domain contains themes that belong to the interpersonal, institutional, community, and policy levels and the practical issues domain themes reflect intrapersonal, interpersonal, institutional, and policy-level factors.

While multiple variations of the framework exist, we employed the 5-layer version to highlight the complex interaction of individual- (i.e., individual characteristics), interpersonal- (i.e., formal and informal social networks and support systems), institutional- (i.e., social institutions with organizational characteristics and rules that govern the institutions), community- (i.e., relationships between organizations, institutions, and informal networks), and policy-level (i.e., laws and policies) factors in shaping one’s vaccination decision-making [45]. This well-established framework has been used extensively in previous research on childhood immunizations, adolescent vaccinations, adult vaccinations, and COVID-19 vaccinations [46–53].”

6. Results: p. 14: “Institutional factors refer to organizational characteristics and rules that govern social institutions.” > In the results section, it might be helpful to clarify that institutional factors include both health facilities and interactions with healthcare staff. This would help readers better understand the connection between this category and your findings.

Response: We have clarified that institutional factors refer both to the institutions themselves as well as the interactions with such institutions in the caption for Figure 1 (lines 262-268).

“Fig 1. Summary of themes and sub-themes by SEM category. Intrapersonal factors represent individual characteristics (e.g., knowledge, attitudes, behaviours, etc.). Interpersonal factors include social networks and support systems. Institutional factors refer to social institutions with organizational characteristics and rules that govern the social institutions, as well as interactions with such social institutions. Community factors encompass relationships between organizations, institutions, and informal networks. Public policy factors include laws and policies.”

7. Results: It would be helpful to indicate the sample size (“n”) to clarify what is meant by terms such as “many participants,” “most participants,” and “almost all study participants.” It might also be useful to identify which reasons were most frequently reported overall and within each SEM category. While not all factors may be actionable, highlighting the most commonly cited reasons could help identify priority areas for improving vaccine acceptance, for example, noting if concerns about vaccine safety were among the most frequently mentioned barriers, or if healthcare provider recommendations were among the strongest facilitators (and how many of the 30 participants reported each).

Response: Thank you for this helpful suggestion. We have identified the most commonly reported motivators (within and across SEM categories) as well as the most important motivator and the most commonly reported and important barrier across SEM categories in lines 492-501.

“Within the different SEM layers, the most commonly reported motivators of vaccination were perceived disease risk to themselves and others (intrapersonal), family and peer norms (interpersonal), healthcare provider recommendation (institutional), church support (community), and government recommendations (policy), among which perceived disease risk to themselves and others was the most commonly reported motivator while healthcare provider recommendation was cited as the most important. Conversely, concerns about vaccine safety was the most common and important reason for not getting vaccinated, consistent with findings previous literature on factors influencing vaccination and vaccine hesitancy among immigrant and migrant populations [7,8,35,36].”

In our study, we conducted a thematic analysis of data, which focuses on identifying patterns of meaning rather than quantifying data (e.g., counting the number of times a theme occurs), which is more applicable for and common in quantitative content analysis. To remain consistent with our qualitative analytic approach, we preserved the wording without indicating sample size.

8. Discussion (p.25): “Consistent with established frameworks of vaccination…” > It would be interesting to clarify in the methods section why Bronfenbrenner’s socio-ecological model was chosen over the more vaccination-specific WHO BeSD framework. Since the findings appear to align with all BeSD domains, I would recommend briefly stating these domains (with their names and short descriptions) and reflecting on how the two frameworks (Bronfenbrenner’s and BeSD) relate to each other and to your findings.

Response: We have clarified the reason for choosing the SEM over a more vaccination-specific model such as the WHO BeSD framework and commented on the alignment between the BeSD framework and the SEM in lines 216-227.

“We chose to present our results using the SEM over more vaccination-specific frameworks such as the World Health Organization’s behavioural and social drivers of vaccination [16], which was conceptualized and developed using the SEM [43], to highlight the interaction of factors across different layers in shaping vaccination decision-making, as well as the importance of higher-level, more structural and systemic factors that impact vaccination decision-making, partially through downstream effects on individual-level determinants of vaccination. Notably, the domains in the BeSD of vaccination are related to and reflect various SEM levels [44]. For instance, the thinking and feeling domain corresponds to the intrapersonal level, whereas the social processes domain contains themes that belong to the interpersonal, institutional, community, and policy levels and the practical issues domain themes reflect intrapersonal, interpersonal, institutional, and policy-level factors.”

9. Discussion (p.25): “…concerns about vaccine safety was the most common reason for not getting vaccinated.” > This finding is well documented in the existing literature. To strengthen the discussion, you could consider adding references to other studies reporting similar results, both general studies on vaccine hesitancy and, ideally, those specifically focused on immigrant populations.

Response: We have included references to studies reporting concerns about vaccine safety as an important/common barrier to vaccination/reason for vaccine hesitancy among migrant/immigrant populations in lines 498-501.

“Conversely, concerns about vaccine safety was the most common and important reason for not getting vaccinated, consistent with findings previous literature on factors influencing vaccination and vaccine hesitancy among immigrant and migrant populations [7,8,35,36].”

10. Discussion re: limitations (p.29-30): I would suggest adding information about potential bias introduced by excluding individuals who were unable to pay for transportation or participate via videoconference (as mentioned in the methods section).

Response: We have included this limitation in lines 592-594.

“Second, we excluded individuals unable to pay for transportation or participate via videoconference, which may have biased the results.”

11. Conclusions (p.30): “Further studies among immigrants of different age groups and from other countries are needed for recommended vaccinations with low uptake rates as vaccination decision-making is context- and vaccine-specific.” > I agree with this statement. However, given the focus of your study, I would suggest being more specific by recommending further research on Korean immigrants across different age groups and in cities beyond Montreal and Toronto, as well as on the most commonly reported reasons for low vaccine uptake and potential strategies to address them based on your findings.

Response: We have revised the conclusion based on your recommendation in lines 619-623.

“Further studies among Korean immigrants of different age groups and in cities beyond Montreal and Toronto on their vaccination decision-making processes, reasons for non-vaccination, and potential strategies to improve vaccination coverage are needed for recommended vaccinations with low uptake rates as vaccination decision-making is context- and vaccine-specific.”

**Reviewer 2**

The study is well-designed and the methodology is sound. The discussion is excellent. Some aspects can be revised to improve the manuscript further. First, tighten the introduction, the paragraph regarding the background of Korean immigration can be reduced into a sentence. Consider adding a comparison of Korean senior vaccination rates versus the national average and other minorities. In my understanding, Koreans have a higher prevalence of vaccination compared to other visible minorities other than others in the East Asian communities but my understanding can be outdated or only applied to COVID-19 vaccines. Table 1 can be made prettier and more condensed. Excellent work relevant and timely for Korean Canadians.

Response: Thank you for your review of our submission. We have removed some details about the background of Korean immigration in the introduction to make the paragraph more concise. The revised background can be found in lines 96-101.

“An older immigrant community that remains heavily overlooked and understudied with respect to vaccination is the Korean immigrant population, one of the largest racial minoritized groups in Canada according to the 2021 Census [20]. This population continues to grow rapidly as well. Between 2001 and 2021, the number of individuals who self-identify as Korean more than doubled, increasing from 101,000 to 218,000, of which over 60% were born in Korea [20,21].”

We have also added some comparisons of vaccination coverage among older adult Koreans versus other minoritized groups in lines 102-112.

“According to a study conducted using 2003-2009 Canadian Community Health Survey data, Korean adults aged 65 years and older reported lower, although not statistically significant, coverage of influenza vaccination than those of other ethnic groups with the exception of Latin American, West Asian/Arab, and Black older adults [22]. This was the only study in the Canadian context that reported vaccination coverage among Korean older adults, but was not specific to Korean immigrant older adults. Similarly, a study conducted using 2009-2012 REACH US Risk Factor Survey reported that Korean American adults aged 50 years and older reported lower influenza vaccination coverage than those of other ethnic groups with the exception of Non-Hispanic Black adults, and that Korean adults aged 65 years and older reported lowest coverage of pneumococcal vaccination among population groups analyzed [23].”

Lastly, we have edited Table 1 to remove double-spacing and condense the table.
